# The Genuine Works of Hippocrates

**Published:** 1886-08

**Authors:** 


**The Genuine Works of Hippocrates,** translated from the Greek, with a preliminary and annotation, by FRANCIS ADAMS, L. L. D., Surgeon. In two volumes. Vol. I. New York: Wm. Wood & Co.

